# Dynamic functional connectivity profile of the salience network across the life span

**DOI:** 10.1002/hbm.25581

**Published:** 2021-07-26

**Authors:** William Snyder, Lucina Q. Uddin, Jason S. Nomi

**Affiliations:** ^1^ Program in Neuroscience Bucknell University Lewisburg Pennsylvania; ^2^ Department of Psychology University of Miami Coral Gables Florida; ^3^ Neuroscience Program University of Miami Miller School of Medicine Miami Florida

**Keywords:** aging, brain development, brain dynamics, flexibility, insular cortex, metastability, midcingulo‐insular network

## Abstract

The insular cortex and anterior cingulate cortex together comprise the salience or midcingulo‐insular network, involved in detecting salient events and initiating control signals to mediate brain network dynamics. The extent to which functional coupling between the salience network and the rest of the brain undergoes changes due to development and aging is at present largely unexplored. Here, we examine dynamic functional connectivity (dFC) of the salience network in a large life span sample (*n* = 601; 6–85 years old). A sliding‐window analysis and *k*‐means clustering revealed five states of dFC formed with the salience network, characterized by either widespread asynchrony or different patterns of synchrony between the salience network and other brain regions. We determined the frequency, dwell time, total transitions, and specific state‐to‐state transitions for each state and subject, regressing the metrics with subjects' age to identify life span trends. A dynamic state characterized by low connectivity between the salience network and the rest of the brain had a strong positive quadratic relationship between age and both frequency and dwell time. Additional frequency, dwell time, total transitions, and state‐to‐state transition trends were observed with other salience network states. Our results highlight the metastable dynamics of the salience network and its role in the maturation of brain regions critical for cognition.

## Graphical abstract

Here, we examine dynamic functional connectivity (dFC) of the salience network in a large life span sample (*n* = 601). A sliding‐window analysis and *k*‐means clustering revealed five states of dFC formed with the salience network, characterized by either widespread asynchrony or different patterns of synchrony between the salience network and other brain regions. Life span trends in the frequency, dwell time, and transitions of these states highlight the role of metastability maturation in cognitively important brain regions.

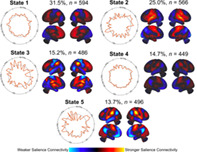

## 1 INTRODUCTION

The salience or midcingulo‐insular network, with key nodes in the anterior insula (AI) and anterior cingulate cortex (ACC), is known for its role in directing attention to relevant or “salient” stimuli and is widely implicated in cognitive and affective processing (Uddin, 2015). The connections between the insula and other brain regions enable salience network functions, such as connections with frontal brain regions facilitating higher‐order cognition (Deen, Pitskel, & Pelphrey, 2011; Uddin, Nomi, Hébert‐Seropian, Ghaziri, & Boucher, 2017). Meta‐analyses have demonstrated that the dorsal AI (dAI) in particular is active during a variety of high‐level cognitive tasks (Kurth, Zilles, Fox, Laird, & Eickhoff, 2010; Uddin et al., 2017; Uddin, Kinnison, Pessoa, & Anderson, 2014; Yeo et al., 2015). The dAI and ACC will therefore be useful candidate regions to investigate the neural correlates of cognitive maturation. Although previous neuroimaging studies have identified the static (e.g., Fair et al., 2007; Uddin, Supekar, Ryali, & Menon, 2011) and dynamic (Nomi et al., 2016) functional connections of the dAI and ACC in child and adult populations, the life span trajectories of dynamic properties for these functional connections are still unclear.

Functional magnetic resonance imaging (fMRI) can characterize life span trajectories of large‐scale brain function using metrics like static and dynamic functional connectivity (dFC). In general, functional connectivity indexes the synchrony, coherence, or correlation of the blood‐oxygen level‐dependent (BOLD) signal between brain regions (Friston, 1994). Observable in resting‐state fMRI data, the functional connections present in the brain reflect the intrinsic organization of brain networks that are also employed during active cognitive and behavioral states (Smith et al., 2009). Static functional connectivity can describe the average synchrony between brain regions by correlating their respective BOLD signals over the duration of a full fMRI scan. dFC can identify more transient fluctuations of functional connectivity that are often masked by static connectivity methods. For example, by splitting the fMRI scan into subsets, or “sliding windows” (Allen et al., 2014), changes in the strength of functional connections over time can be tracked. Multiple changes in functional connectivity strength often coincide, forming separable patterns or dynamic states present for short periods of time (Hutchison et al., 2013; Zhou, Zhang, Feng, & Lo, 2019). Therefore, dynamic approaches can supplement our understanding of how static functional connections change throughout life by also characterizing moment‐to‐moment changes in the brain.

The static and dFC between brain regions evolve throughout the life span. In contrast to white matter tracts (Hermoye et al., 2006; Mukherjee et al., 2002) and sulcal shapes (Dubois et al., 2008; Nishikuni & Ribas, 2013) that are largely laid down early in life, large‐scale functional brain networks markedly remodel throughout all stages of life (Bagarinao et al., 2019; Faghiri, Stephen, Wang, Wilson, & Calhoun, 2019; Grayson & Fair, 2017; Gu et al., 2015; Supekar, Musen, & Menon, 2009; Váša et al., 2020; Vij, Nomi, Dajani, & Uddin, 2018; Xia et al., 2019). Overall trends in static functional connectivity demonstrate a U‐shaped curve relating between‐network connectivity and age, while within‐network connectivity follows an inverse U‐shaped curve (Betzel et al., 2014). Different graph theoretic approaches to static connectivity reveal different topological changes across the life span (Bullmore & Sporns, 2009; Cao et al., 2014; Luo et al., 2020; Zuo et al., 2010) and have demonstrated region‐specific developmental trajectories. In contrast, whole‐brain dynamics are emphasized in life span analyses utilizing dFC. Parsing region‐specific dynamics, however, can sometimes better capture behaviorally relevant network changes (Battaglia et al., 2020; Lombardo et al., 2020). A focused analysis of the salience network/midcingulo‐insular network would promote an understanding of how networks important for cognitive and executive control change throughout the life span.

A dFC analysis will be useful to capture the functional diversity and network‐switching properties of the salience network. Meta‐analysis of the dAI demonstrates its coactivation partners are involved in a variety of behaviors (Uddin et al., 2014). This functional diversity was also revealed in a dFC analysis demonstrating the dAI engage in highly variable brain states (Nomi et al., 2016). Notably, connections between the dAI and frontal, medial‐frontoparietal, and temporal regions were differentiated in the dynamic states. The separable states of connectivity are thought to subserve flexible network switching. The dAI and ACC are posited to mediate the engagement of the lateral‐frontoparietal executive control and medial‐frontoparietal default mode networks (Uddin, 2015; Uddin et al., 2017). Cognitively demanding tasks lead to increased activity in the salience/midcingulo‐insular and lateral‐frontoparietal executive control networks and decreased activity in the medial‐frontoparietal default mode network (Menon & Uddin, 2010). dFC analyses of resting‐state fMRI can offer insight into how intrinsic properties of the brain support these dynamics throughout the life span.

Here, we perform a dFC analysis of salience network maturation in a cross‐sectional life span data set. We leveraged the large sample of resting‐state fMRI in the Nathan Kline's Institute database to investigate trends in neurotypical individuals. In a cohort of 601 subjects aged 6–85, we identified states of dFC associated with the dAI and ACC using a sliding‐window approach. By relating properties of these dynamic states with age, we present a thorough characterization of salience network dFC maturation. This work will provide a useful benchmark against which to evaluate deviant maturational trajectories of this critical brain network.

## 2 MATERIALS AND METHODS

### 2.1 Life span cohort

**FIGURE 1:**
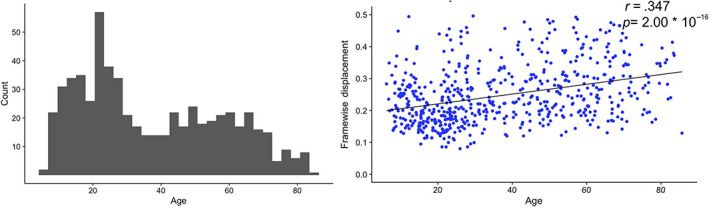
Sample age distribution and head motion. The 601 subjects retained from the NKI database sufficiently spanned ages 6–85 (left), allowing for a life span analysis. Subjects' head motion (right) during fMRI sessions had a positive, linear relationship with age, so head motion was controlled for in subsequent analyses. fMRI, Functional magnetic resonance imaging

Resting‐state scans were obtained from the Enhanced Nathan Kline Institute Rockland Sample (http://fcon_1000.projects.nitrc.org/indi/enhanced/). Subjects with a current Axis‐I DSM diagnosis or with head motion greater than 0.5 mm framewise displacement (FD) in subsequent processing were excluded from the study. Additionally, one subject was excluded for absent information regarding sex designation. The remaining cohort of 601 subjects (Figure 1) was used in the final analysis. A linear regression with age and sex as independent variables and FD as the dependent variable showed a significant effect of age (*p* = 2 * 10^−16^), thus FD was controlled for in subsequent analyses.

### 2.2 fMRI data preprocessing

Each subject had one 10‐min resting‐state fMRI scan collected using a Siemens Trio 3.0T scanner (multiband factor of 4, 23 mm, 40 interleaved slices, TR = 1.40 s, TE = 30 ms, flip angle = 65°, FOV = 224 mm, 404 volumes). The first five volumes were removed to ensure magnetic stability was reached for each scan. The data were despiked (AFNI's 3dDespike “new” algorithm), realigned, normalized (directly to 3 mm MNI space using a SPM template), and smoothed (AFNI's 3dBlurToFWHM 6 mm) using the Data Processing Assistant for Resting‐State fMRI (DPARSF‐A). Slice‐timing correction was not performed as it has minimal impact on BOLD functional connectivity and variability (Wu et al., 2011), and is generally not employed in data acquired with multi‐band sequences (e.g., Zachary et al., 2021; Roye et al., 2020; Vieira, Rondinoni, & Garrido Salmon, 2020). FMRIB's independent component analysis (ICA) based Xnoiseifier (FIX) was then used to automatically classify noise and non‐noise components in each single subject ICA and subsequently regress out noise components. The FIX classifier was trained using hand‐classification of 24 subjects randomly selected across 10‐year age brackets. Additionally, the 24 subjects consisted of subjects with high and low average FD in each age bracket in order to ensure all age ranges and head motion possibilities were equally represented in the automatic classification.

### 2.3 High‐model order ICA

Following previous work (Allen et al., 2014; Nomi et al., 2016; Nomi et al., 2017), we performed a spatial group ICA on the preprocessed data using the Group ICA of FMRI Toolbox (GIFT) infomax algorithm. Spatial ICA maximizes the statistical independence of spatial image components, allowing for extraction of component time courses from spatially distinct brain regions. High‐model order ICA with a set 100 components decomposes images into brain regions that comprise larger brain networks spanning cortical, subcortical, and cerebellar brain regions (Allen et al., 2014; Nomi et al., 2016). The ICA was repeated 20 times with the Icasso algorithm to identify stable, reproducible components. The group ICA (GICA1) back‐reconstruction (Calhoun et al., 2001) algorithm generated subject specific spatial maps and time courses from each independent component from the group ICA.

After removal of noise‐related components (head motion artifacts, white matter, cerebral spinal fluid, etc.) by visual inspection, we retained 65 non‐noise components. The noise‐related components were characterized by peak activations in white matter or ventricles, resembling head motion, or by excessive high frequency information in time courses. The component of interest included both the dAI and the ACC indicating that the time courses in these areas were tightly coupled. The high synchrony of these two regions resulted in a single component representing the key nodes of the salience network (Uddin, Yeo, & Spreng, 2019).

### 2.4 Post‐processing

The time courses for each non‐noise component output by GIFT were triple detrended, despiked, regressed against the global average signal of non‐noise ICs and the Friston 24 head motion parameters estimated by DPARSF‐A preprocessing, and finally band‐pass filtered (0.023–0.1 Hz). Despiking was conducted using MATLAB code from the GIFT toolbox based on AFNI's 3dDespike algorithm that replaces outliers within each time series greater than 3 *SD* with data based on the mean *SD* of the time‐series. The high frequency cutoff was chosen to include only BOLD‐related signal fluctuations (Cordes et al., 2001) and is consistent with previous dynamic fMRI analyses. The low frequency cutoff was chosen as the lowest frequency signal possibly present in a sliding window analysis with 44.8 s windows (Leonardi & Van de Ville, 2015).

### 2.5 Salience network dFC and cluster analysis

dFC between all ICs was computed in the GIFT dynamic‐functional network connectivity (d‐FNC) toolbox for subsequent analyses. The d‐FNC toolbox computes dynamic connectivity through a sliding‐window analysis, calculating correlations between ICs within windows of the rs‐fMRI scan. The choice of window size influences the estimates of dFC. Previous research has demonstrated that window sizes between 30 and 60 s (Allen et al., 2014; Hutchison et al., 2013), and notably those at 44 (Yang, Craddock, Margulies, Yan, & Milham, 2014) or 45 s (Allen et al., 2014; Damaraju et al., 2014; Rashid, Damaraju, Pearlson, & Calhoun, 2014) are effective at capturing fluctuations in functional connectivity strength over time. Since our data set had a TR of 1.4 s, we, therefore, chose to use sliding windows spanning 32 volumes, or 44.8 s. We additionally tested window sizes of 1.5× and 2× this window size (67.2 and 89.6 s) to evaluate the robustness of the results across different window sizes (see Data S1). The d‐FNC toolbox additionally convolves the windows with a 3‐sigma Gaussian curve to smooth transitions in connectivity strengths locally between windows. Windowed correlation matrices were regularized with the graphical LASSO method (Varoquaux, Gramfort, Poline, & Thirion, 2010) to minimize within‐window noise. The graphical LASSO method estimates functional connectivity by applying L1 regularization to the inverse covariance matrix, optimizing the lambda parameter separately for each subject (Allen et al., 2014; Damaraju et al., 2014; Nomi et al., 2016; Yang et al., 2014). For each subject, 367 windowed correlation matrices were produced, representing pairwise correlations between 65 brain regions. The connections of interest were the correlations between the salience network (dAI and ACC) and the 64 other ICs that were extracted and Fisher‐z transformed prior to clustering.

Clustering with the *k*‐means method has been successful in identifying separable, functionally differentiated states of dFC. For each subject and for each window, the dFCs between the salience network and 64 other brain regions were represented as a 64‐dimensional vector. *K*‐means clustering would then identify clusters of points in a 64‐dimensional functional connectivity space, with clusters grouping together points with similar patterns of functional connectivity with the salience network. Our sample included 601 subjects and 367 windows per subjects, so 220,567 points in 64‐dimensional space were clustered to identify stereotyped states of connectivity formed with the salience network. Distances between points were computed with the “city‐block” method, which performs well when identifying clusters in a high‐dimensional space (Aggarwal, Hinneburg, & Keim, 2001). The optimal number of clusters (“*k*”) was chosen after running clustering with k values between 2 and 20 and applying the elbow criterion (Allen et al., 2014; Denkova et al., 2019; Nomi et al., 2016; Nomi et al., 2017). The ratio of within‐cluster sum of squared distances to between‐cluster sum of squared distances evaluates the degree to which clusters contain tightly packed points within clusters and distantly separated clusters. The “elbow” in the plot of this metric for each value of *k* identifies the *k* for which robust clusters are found without over‐ or under‐fitting (see Data S1). After determining the optimal *k*, we ran *k*‐means clustering to assign each point (or, window of connectivity with the salience network) a state label.

*K*‐means clustering does not ensure that each resulting brain state represents biologically plausible neural networks that differ from random brain activity. Several methodologies for constructing null models for brain dynamics exist (Miller et al., 2018), with surrogate data sets maintaining first order properties of the BOLD data such as variability (Damaraju et al., 2014; Marshall et al., 2020). These surrogate data sets use the original fMRI time series and phase randomize the signals so that the mean, variability, and autocorrelation are unchanged but the temporal order of the signal is scrambled. By phase‐randomizing our time series according to the procedure in Lancaster et al. (2018) prior to 44.8 s sliding window d‐FNC analysis, we tested such a null model to compare to the original data set's cluster solutions (see Data S1).

### 2.6 Subject state metrics and regression with age

The assignment of dFC states to each window allows for analysis of how these states present within each subject's rs‐fMRI scan. We computed the frequency, dwell time, number of total transitions, and number of transitions between specific states for each subject. Frequency is computed as the proportion that a state occurs in relation to all possible TRs in each subject's rs‐fMRI scan. Dwell time is the average number of consecutive windows a state is instantiated. The total number of transitions is the total number of state switches between consecutive windows. The number of specific state‐to‐state transitions is computed pairwise for each set of possible state transitions (e.g., State 2 to 3, or, State 3 to 2).

Each metric was subjected to multiple linear regression with age, including sex and head motion (FD) as covariates. Quadratic effects were tested by multiple linear regression with each metric and a linear age term, a mean‐centered and squared age term, sex, and head motion. Since some subjects did not enter a given state, a dwell time of zero for that subject and state would inaccurately suggest a low dwell period occurred, when in fact no dwell period can be estimated. To correct for this, regressions with dwell time included only subjects that engaged in a given state, yielding a different subset of subjects for each dwell time regression. A frequency of zero when a subject does not enter a state, however, is still informative to the overall occurrence of that state in a subject, and so all frequency regressions drew upon the whole sample. When assessing significance, Bonferroni correction was applied to correct for multiple comparisons for each state metric.

## 3 RESULTS

### 3.1 ICA

**FIGURE 2:**
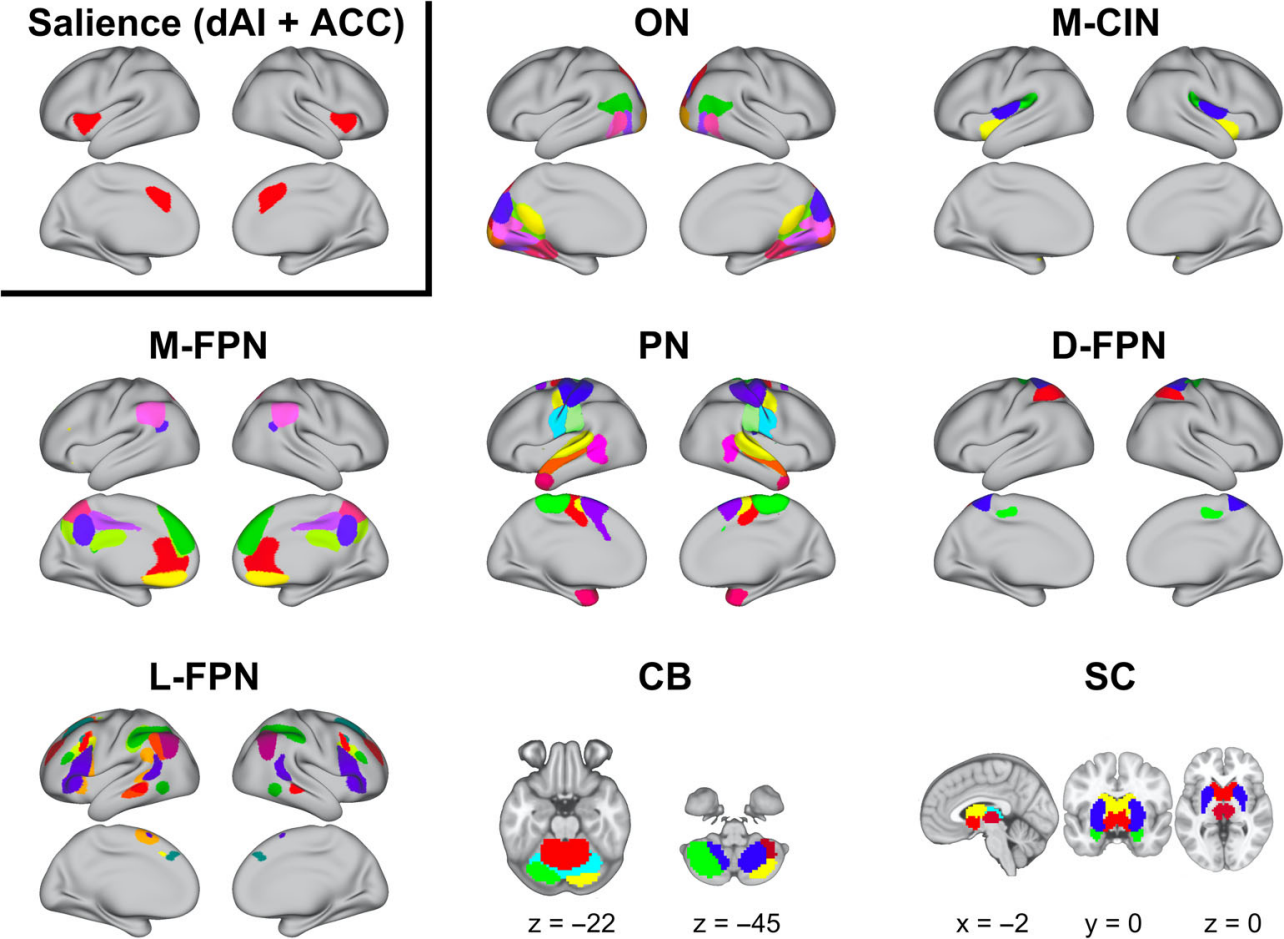
High‐model order ICA parcellation. Sixty‐five non‐noise ICs across eight networks are depicted. Different colors within each network pertain to each IC used in the dFC analysis. The IC for the salience network is highlighted (top left) since dFC was evaluated between this region and each of the 64 other ICs. CB, cerebellum; dFC, dynamic functional connectivity; D‐FPN, dorsal‐frontoparietal network; L‐FPN, lateral‐frontoparietal network; M‐CIN, midcingulo‐insular network; M‐FPN, medial‐frontoparietal network; ON, Occipital Network; PN, pericentral network; SC, subcortical

The 65 non‐noise ICs retained from the 100 component ICA represented distinct brain regions in cortical, subcortical, and cerebellar networks. The ICs were grouped into sensorimotor, visual, default mode, salience, temporal, central executive, frontal, cerebellar, parietal, and subcortical networks for visualization (Figure 2). The parcellation resembled previous works using a high‐model order ICA (Allen et al., 2014; Nomi et al., 2016).

### 3.2 States of salience network dFC

**FIGURE 3:**
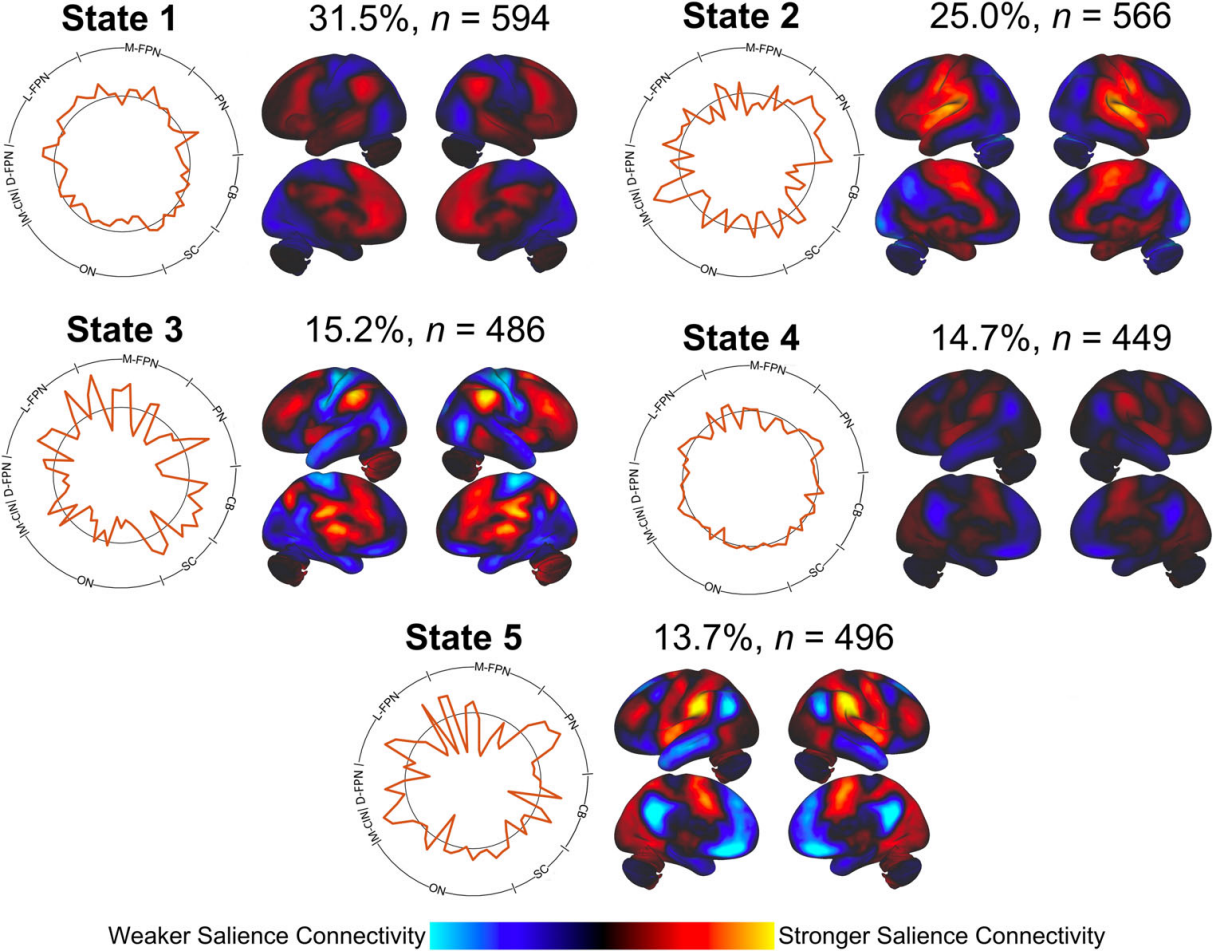
States of salience network dynamic functional connectivity (dFC). Cluster analysis revealed five states of salience network functional connectivity. The percentage of total states and the number of subjects that entered into each state are shown above each state brain projection. Profiles of the transient connectivity states are shown in both the polar plots and brain projections. The magnitude of points on the polar plot indicates the strength of dFCs between the salience network and brain regions across eight networks. Positive values on the polar plots extend past the black ring. ICs were scaled by their corresponding magnitudes in the polar plot and summed together to depict the functional connectivity state projected back on the brain. The color scale (arbitrary units) maps the strength of salience network functional connectivity across the brain. States exhibited different patterns of synchrony or lack of synchrony with the salience network

We present results for dFC analyses using 44.8 s sliding windows. Highly similar results for analyses with 67.2 s and 89.6 s windows can be found in the Data S1. An optimal *k* = 5 from the elbow criterion led to the identification of five states of salience network dFC (Figure 3). State 1 was characterized by near‐zero correlations between the salience network and most other brain regions, with low synchrony observed with M‐FPN and additional frontal regions. State 2 was characterized by salience network functional connectivity with sensorimotor, insular, and medial frontal brain regions. State 3 was characterized by salience network functional connectivity with lateral‐frontoparietal, medial‐frontoparietal, and subcortical brain regions. State 4, similar to State 1, was characterized by predominantly near‐zero correlations between the salience network and most other brain regions. State 5 was characterized by sensorimotor, parietal, insular, and medial visual brain regions.

The same set of states was found in both the 67.2 s (Figure S3) and 89.6 s (Figure S8) sliding window analyses. The proportion of these states observed in the data set varied by the length of the sliding window, with longer window sizes leading to more balanced proportions of states being observed. When testing a null model constructed from phase‐randomized time series with a five‐state cluster solution, all states consisted of near‐zero correlations with the salience network and all other brain regions (Figure S11). This lack of any discernible patterns in a surrogate phase‐randomized data set suggests the validity of salience network dFC patterns observed in our original data set.

### 3.3 Associations between dynamic metrics and age

**FIGURE 4:**
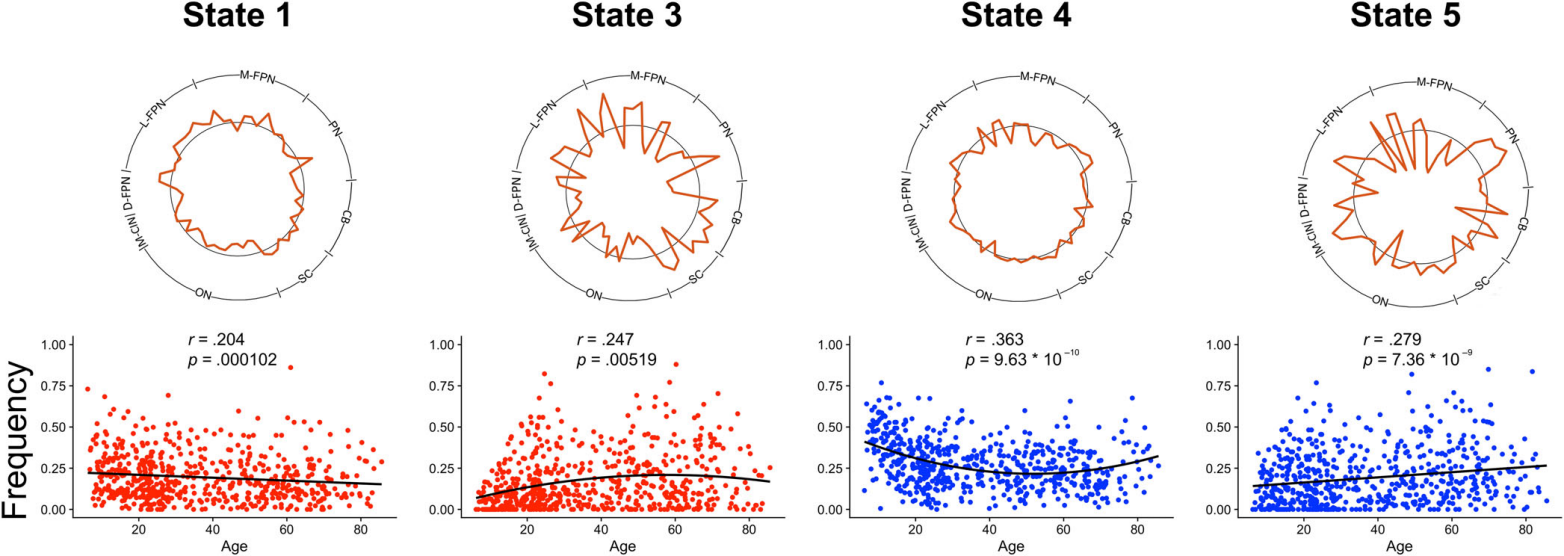
Associations between state frequency and age. Positive (blue) and negative (red) linear and quadratic trends between subject age and state frequency were observed. Each relationship was significant after Bonferroni correction (*p* < .01)

**FIGURE 5:**
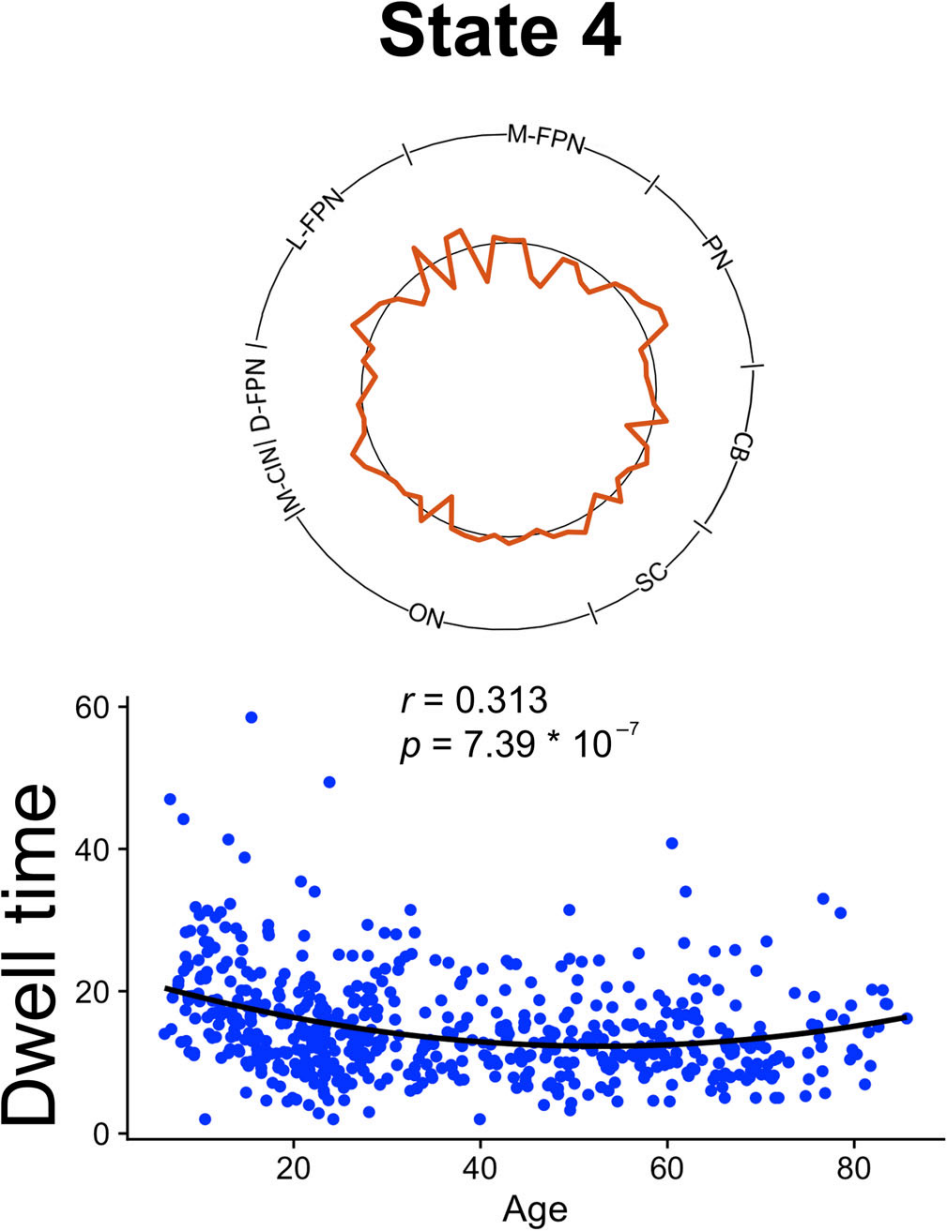
Association between state dwell time and age. A positive quadratic relationship between state dwell time and age was observed, significant after Bonferroni correction (*p* < .01)

**FIGURE 6:**
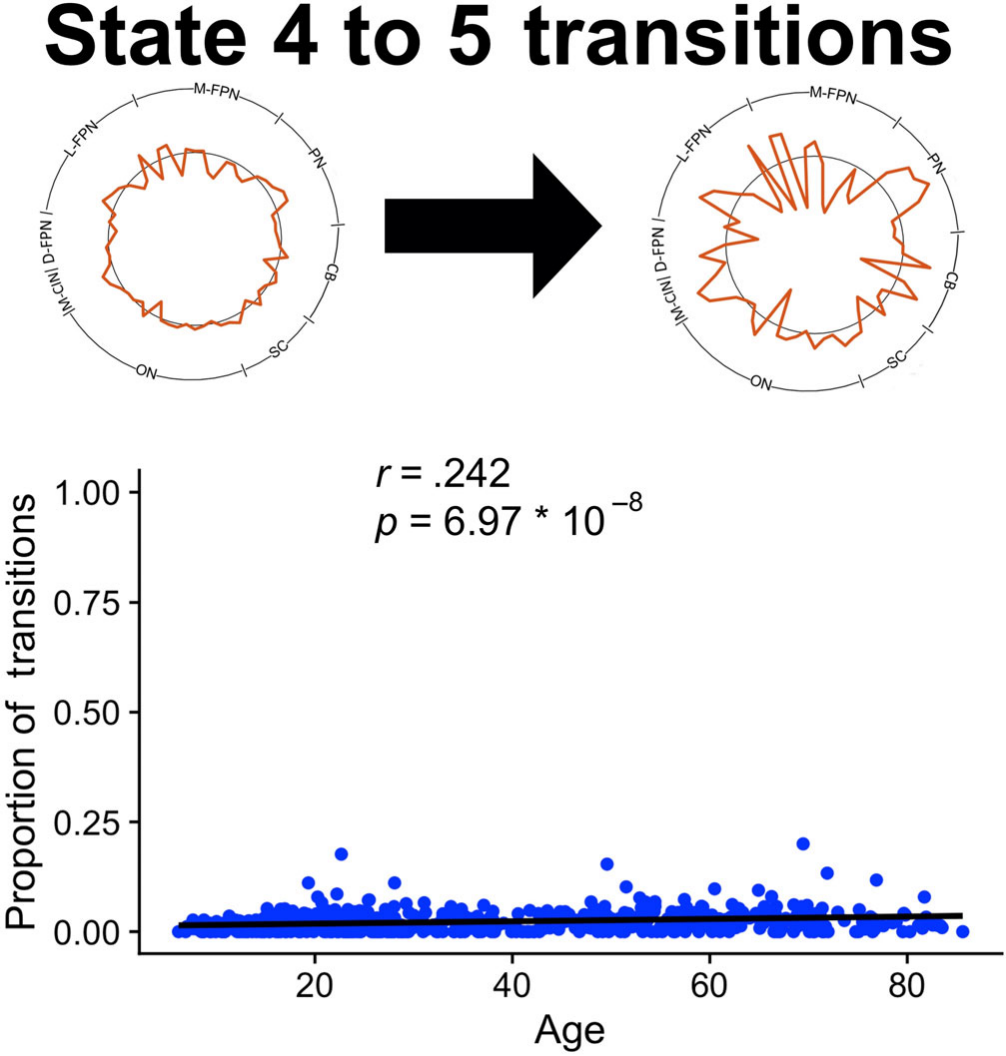
Association between state transitions and age. No significant total state transition relationships were observed, but a specific state‐to‐state transition association was revealed. The proportion of State 4 to State 5 transitions had a positive linear relationship with age. Significance survived Bonferroni correction for the 20 possible state transitions possible amongst five states (*p* < .0025)

Linear regressions revealed linear and quadratic effects of age on dynamic metrics. Significant (Bonferroni corrected, *p* < .01) relationships between age and frequency or dwell time are depicted in Figures 4 and 5, respectively. The significant relationships between age and total state transitions (*p* < .05) as well as between age and state‐to‐state transitions (Bonferroni corrected, *p* < .0025) are depicted in Figure 6. All *p*‐values presented are uncorrected and survive these adjusted thresholds. State 1 frequency was negatively correlated with age (*p* = 0.000102). State 3 frequency was negatively correlated with squared age (*p* = .00519). State 4 frequency and dwell time were both positively correlated with squared age (*p* = 9.63 * 10^−10^, *p* = 7.39 * 10^−7^, respectively). State 5 frequency was positively correlated with age (*p* = 7.36 * 10^−9^). No association was found between total transitions between any states for each subject and age. The frequency of transitions specifically from State 4 to State 5 was positively correlated with age (*p* = 6.97 * 10). Qualitatively similar results were found with the analogous states in the 67.2 s and 89.6 s sliding window analysis. However, one frequency trend and the transition trend did not reach significance in the 89.6 s sliding window analysis.

## 4 DISCUSSION

We assessed the maturation of dFCs between the salience network and the whole brain using a sliding window approach (Allen et al., 2014). In a cohort of 601 neurotypical subjects aged 6–85, we found that individuals transitioned amongst five states of salience network functional connectivity during the course of a 10‐min resting state fMRI. The states were differentiated by the brain regions whose BOLD signal was transiently synchronized with the salience network. We evaluated the frequency of each state, the dwell time of each state, total transitions, and state‐to‐state transitions as a function of participant age. Significant relationships between state frequency and age were observed for all states, in addition to relationships between certain states' dwell times and transitions.

State 4, characterized by asynchrony between the salience network and all other brain regions, demonstrated the greatest age‐related associations. State 4 demonstrated a U‐shaped association with frequency and dwell time in addition to a linear positive association with state transitions. The four other states exhibited synchrony between the salience network and multiple other brain regions. Additional trends between the frequency of these states and age were observed. The states of salience network dFC and their relationships with age were also found to be robust to choice of sliding window size.

One way to interpret the results is to consider multiple dFC states' trends simultaneously. For example, the strong U‐shaped frequency trends with State 4 could have driven the weaker, mirrored inverse U‐shaped trends with State 3 frequency. Since State 1 generally decreases in frequency with age while State 5 generally increases in frequency with age, these concurrent changes could reflect a shift toward increasing salience network connectivity with visual and somatosensory regions and decreasing connectivity with medial frontal regions over the life span. This appears to contrast static functional connectivity evidence of increased M‐FPN and salience network connectivity later in life (Ferreira et al., 2016; Malagurski, Liem, Oschwald, Mérillat, & Jäncke, 2020). However, it is difficult to compare trends of individual states of dFC to static functional connectivity results as all dFC trends contribute to overall static functional connectivity trends.

Reconciling a variety of trends is often a challenge for studies piecing together the multi‐factorial process of brain development. A review of these topological changes during senescence (Naik, Banerjee, Bapi, Deco, & Roy, 2017) suggested metastability will best account for multiple life span trends in brain dynamics. Metastability is a network's ability to alternate between states of phase synchrony and asynchrony. In our analysis, the asynchrony in State 4 and patterns of synchrony in the other four states reflect the metastable dynamics of the salience network. We will focus our discussion on this metastability and its implications for cognitive maturation.

### 4.1 Maturation of metastable salience network dynamics

Tognoli and Kelso (2014) describe metastability as the balance between phase‐synchronized and phase‐scattered brain states. Noise can drive the brain out of locked states of phase‐synchronization, promoting switching to other phase‐synchronized states by first entering a state of temporary asynchrony. Neuroscience literature has largely emphasized the investigation of phase‐synchronized states for their ability to predict distinct behaviors. However, there is increasing realization that phase‐scattered states and meta‐stability need to be incorporated in models of complex behaviors, especially those that demand dynamic network interactions. The salience/midcingulo‐insular network is known for its role in network switching, particularly between the medial‐frontoparietal and lateral‐frontoparietal network (Uddin, 2015; Uddin et al., 2019). Metastability may therefore be a useful framework to interpret the maturation of the dynamics underlying network switching.

Our results are consistent with some of the predictions from Naik et al.'s (2017) model of life span metastability trends. Naik et al. (2017) predicted high‐functioning adults would exhibit increased metastability to compensate with structural degradation of brain networks during senescence. In this model, the increased between‐network functional connectivity and decreased within‐network functional connectivity observed in old age can then be reframed as increased meta‐stability decoupling within‐network signals in favor of network switching. Given the life span trends of between and within‐network connectivity (Betzel et al., 2014), metastability would be expected to follow a U‐shaped curve with respect to age. Specifically, a U‐shaped curve relating the frequency or dwell time of an asynchronous state would be expected to be observed alongside a U‐shaped curve for the frequency of state transitions, likely driven by the presence of the asynchronous state. We observed this U‐shaped curve with respect to metastability of the salience network through the frequency and dwell time of State 4, with some evidence suggesting its role in state switching.

Multiple approaches to extract dynamic brain states have led to similar conclusions. Preliminary work using a Hidden Markov Model demonstrated that a mean‐activation state, analogous to an asynchronous state that promotes metastability, followed a U‐shaped curve relating the state's frequency with age (Chen, Wang, & Liu, 2020). Two other studies using a sliding‐window dFC approach found positive, linear correlations between metastability promoting brain states and age. One of the studies analyzed the NKI database, finding a state with weak functional connectivity positively, linearly correlated with age (Chen et al., 2019). Another study found the same result with a state characterized by widespread negative functional connectivity (Xia et al., 2019). The aforementioned studies extracted whole‐brain functional connectivity states, whereas we focused on the states formed with the salience network. Smaller sample sizes and more adult‐focused subject age ranges could have also obscured quadratic trends in other works. Previous works observed linearly increasing network switching with age (Chen et al., 2019; Xia et al., 2019), similar to that seen in State 4 to State 5 transitions. Notably, this modest linear increase does not mirror the expected U‐shaped aging trends expected to be paralleled by that of the U‐shaped frequency and dwell time trends for the asynchronous State 4. Further research may wish to explore how asynchronous states identified in these analyses of intrinsic network dynamics shape network shifting and behavior.

Task‐based fMRI studies and behavioral evidence highlight the cognitive importance of metastability. For example, older subjects switch between networks more often than younger subjects during a memory task, although both groups performed equally well on the task (Schlesinger et al., 2017). The study supports Naik et al.'s (2017) hypothesis that metastability can help older adults maintain cognitive performance despite decline in brain structure. Nomi et al. (2017) found that the frequency of an asynchronous brain state during resting state fMRI predicted performance on executive function, specifically for tasks that require flexible cognition. This work underscores the connection between neural flexibility and cognitive flexibility, lending metastability a potential role in the meta‐control of behavior.

Meta‐control is the ability to switch between heuristic approaches to problem solving, and efficient problem solving involves the balance between persistent strategies and strategy switching. Behavioral differences in meta‐control between groups (Nassar & Troiani, 2020) could stem from differences in neural flexibility or variability (Dajani & Uddin, 2015; Grady & Garrett, 2014; Nomi et al., 2017) mediated by metastable brain dynamics. The normative developmental trajectories of salience network brain dynamics presented in our analysis can provide a basis to understand deviations from it that relate to atypical cognitive flexibility in psychiatric and neurological conditions (Uddin, 2021).

### 4.2 Limitations

State based analyses of dFC using high‐model order ICA are well precedented in the literature but are not without limitations. High‐model order ICA tends to only separate components by hemisphere in lateral‐frontoparietal regions. Higher order ICA is unlikely to meaningfully divide the salience network either by hemisphere or by dividing between the dAI and ACC. Thus, we could not examine the dAI or ACC in isolation using an ICA seed methodology. Given our life span cohort with likely variable brain morphology and functional topography, we chose the ICA approach in order to estimate brain activity while also accounting for individual differences in network topography with ICA back‐reconstruction methods. Future work may choose to use a priori region‐of‐interest approach if the constituent regions of the salience network are of particular interest.

Biases in sample characteristics should also be considered. The NKI database does not have a uniform distribution for subject age, with slightly greater representation of younger (<30 years) subjects. While the large sample size effectively captures brain dynamics across the life span, regressions could better predict data in these age ranges.

### 4.3 Conclusions

In sum, we find that dynamic states of functional connectivity formed with the salience network substantially evolve throughout the human life span. The most pronounced changes in the frequency and dwell time of an asynchronous state point to metastable dynamics as an end of maturational processes within cognitively important brain regions, such as the dAI and ACC. These trajectories may help contextualize cognitive development in terms of the dynamic phenomena underlying complex cognitive processes.

## CONFLICT OF INTEREST

The authors declare no potential conflict of interest.

## Supporting information

**AppendixS1.** Figures.

## Data Availability

Data were retrieved from the open access Enhanced Nathan Kline Institute Rockland Sample (http://fcon_1000.projects.nitrc.org/indi/enhanced/).
