# Supplementary material for: Dynamic functional connectivity profile of the salience network across the life span
Source: Hum Brain Mapp. 2021 Jul 26;42(14):4740–9. doi: 10.1002/hbm.25581 (PMC8410581; doi:10.1002/hbm.25581)
Supplement: Supplementary file 1 — AppendixS1. Figures. [file HBM-42-4740-s001.docx]

**Supplementary Materials**

Dynamic functional connectivity profile of the salience network across the lifespan

William Snyder^1^, Lucina Q. Uddin^2,3^, Jason S. Nomi^2*^

^1^Program in Neuroscience, Bucknell University, Lewisburg, PA, USA

^2^Department of Psychology, University of Miami, Coral Gables FL, USA

^3^Neuroscience Program, University of Miami Miller School of Medicine, Miami, FL, USA

*Correspondence should be addressed to:

Jason S. Nomi

University of Miami

P.O. Box 248185-0751

Coral Gables, FL 33124

Email: jxn131@miami.edu

Phone: 305-284-3273

**Contents:**

**Supplementary Figure 1.** Elbow plot for k-means clustering of 44.8s window results.

**Supplementary Figure 2.** Elbow plot for k-means clustering of 67.2s window results.

**Supplementary Figure 3.** States of salience network dFC with 67.2 s sliding windows.

**Supplementary Figure 4.** Associations between state frequency and age under 67.2s windows.

**Supplementary Figure 5.** Associations between state dwell time and age under 67.2s windows.

**Supplementary Figure 6.** Associations between state transitions and age under 67.2s windows.

**Supplementary Figure 7.** Elbow plot for k-means clustering of 89.6s window results.

**Supplementary Figure 8.** States of salience network dFC with 89.6 s sliding windows.

**Supplementary Figure 9.** Associations between state frequency and age under 89.6s windows.

**Supplementary Figure 10.** Associations between state dwell time and age under 89.6s windows.

**Supplementary Figure 11.** States of salience network dFC with phase-randomized surrogate data and 44.8s sliding windows.

**Supplementary Figure 1.** Elbow plot for *k*-means clustering of 44.8s window results. The cluster validity index was calculated as the ratio of within-cluster sum of squared distances to between-cluster sum of squared distances between clustered points. Clusters of size *k* = 2 through *k* = 20 were evaluated.
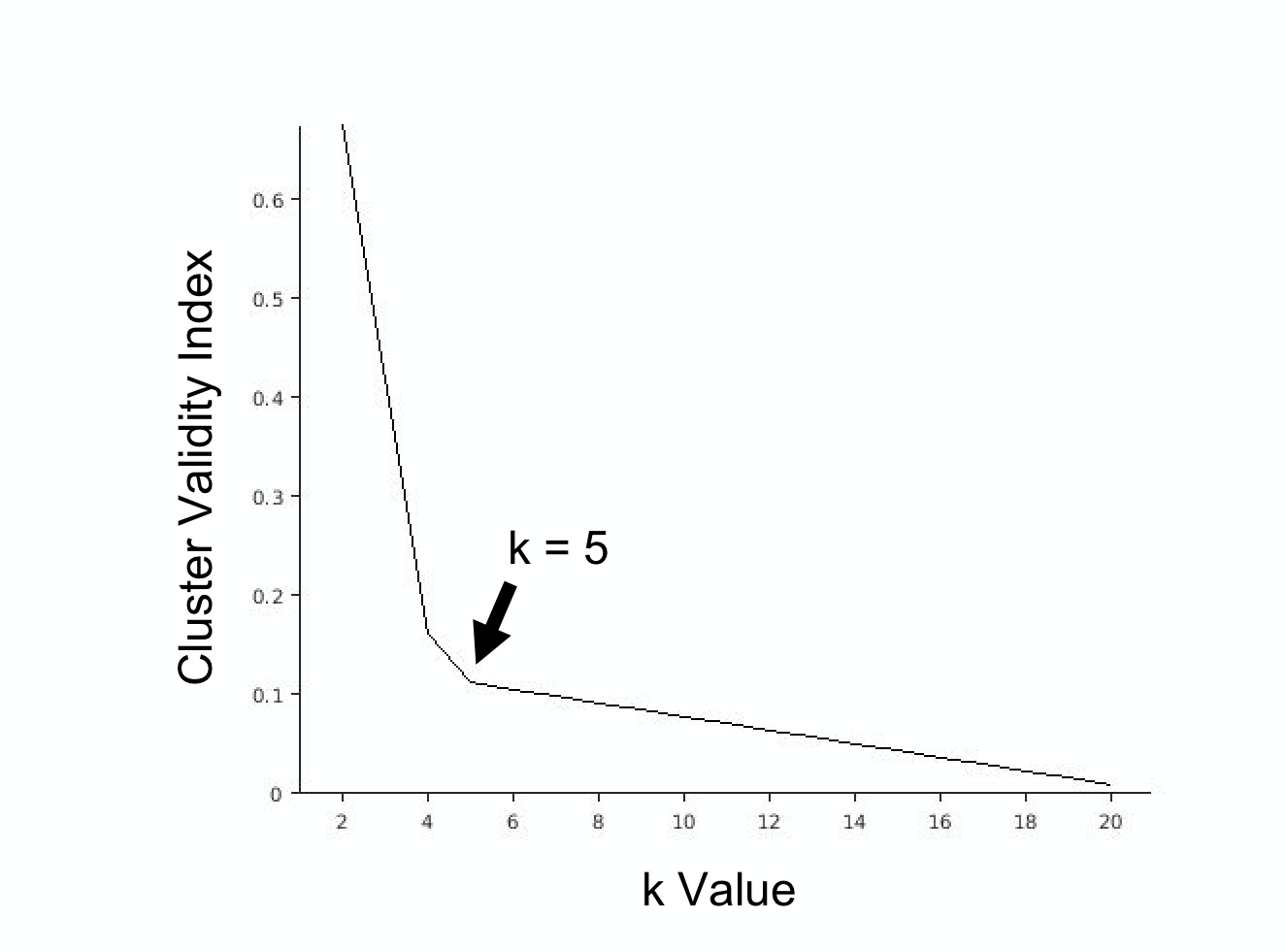

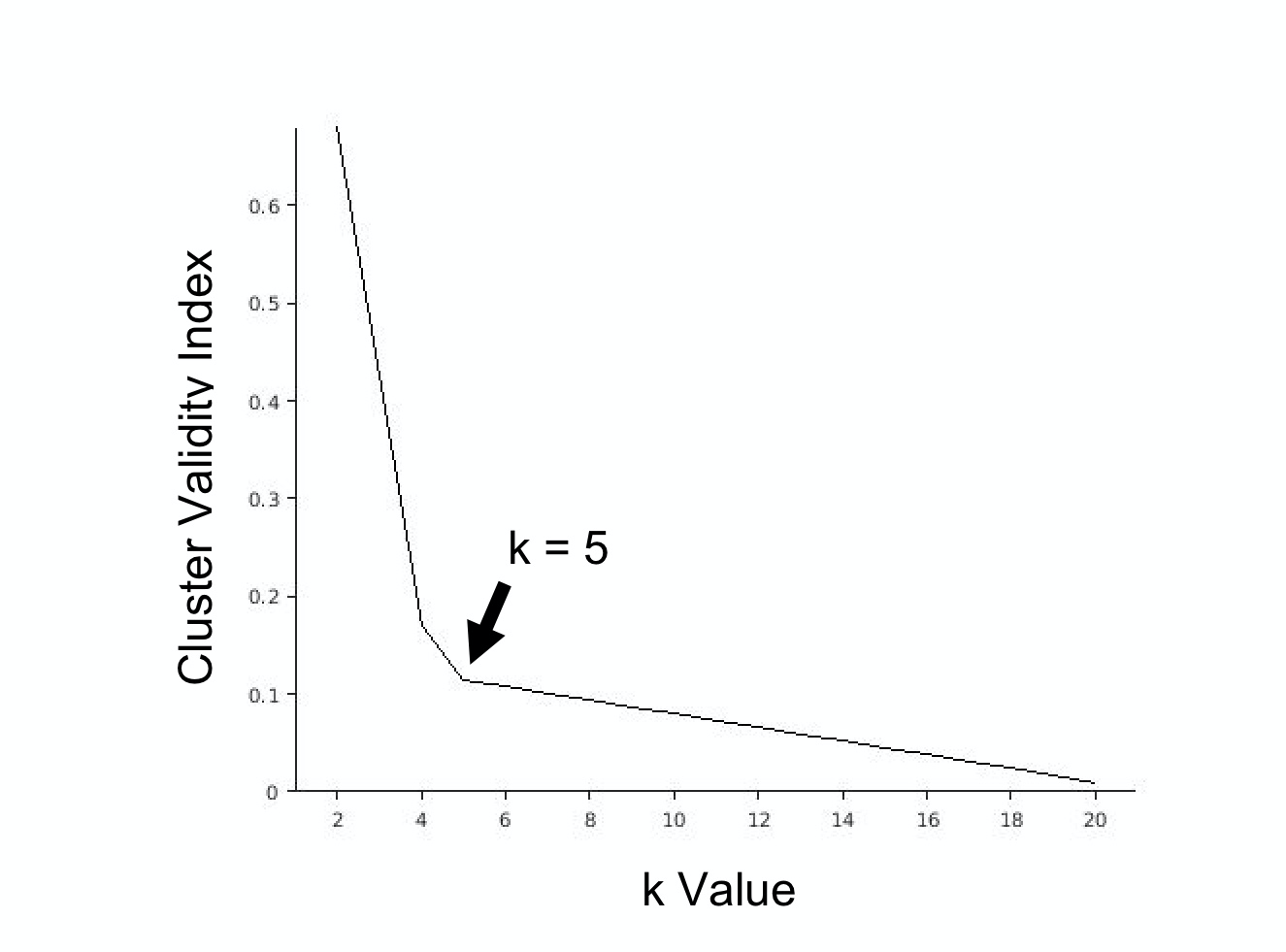


**Supplementary Figure 2.** Elbow plot for *k*-means clustering of 67.2s window results.


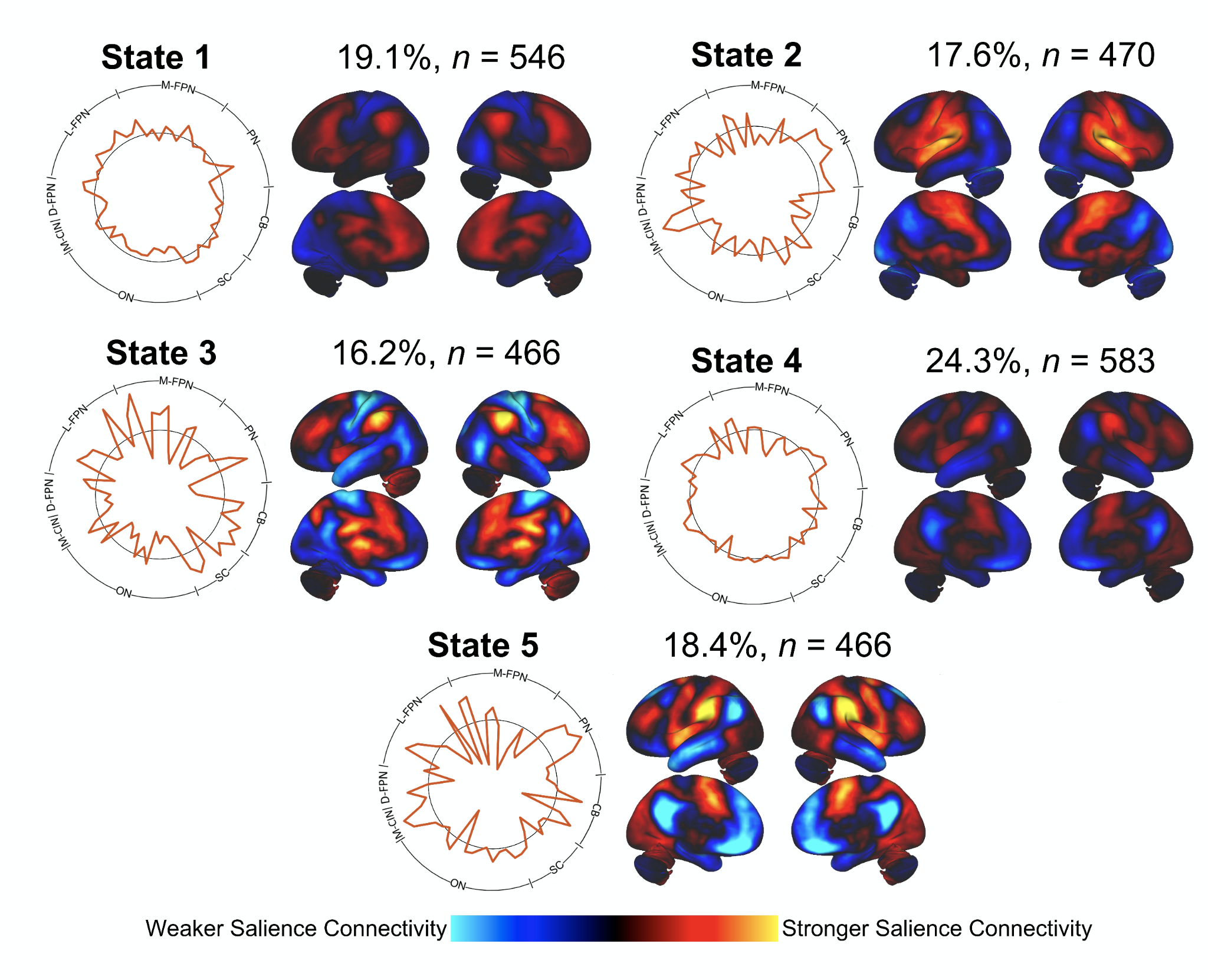


**Supplementary Figure 3.** States of salience network dFC with 67.2 s sliding windows. States revealed were highly similar to those found using 44.8s sliding windows, with two states having low/near-zero correlations and the other states exhibiting the same patterns seen in states using 44.8s windows.


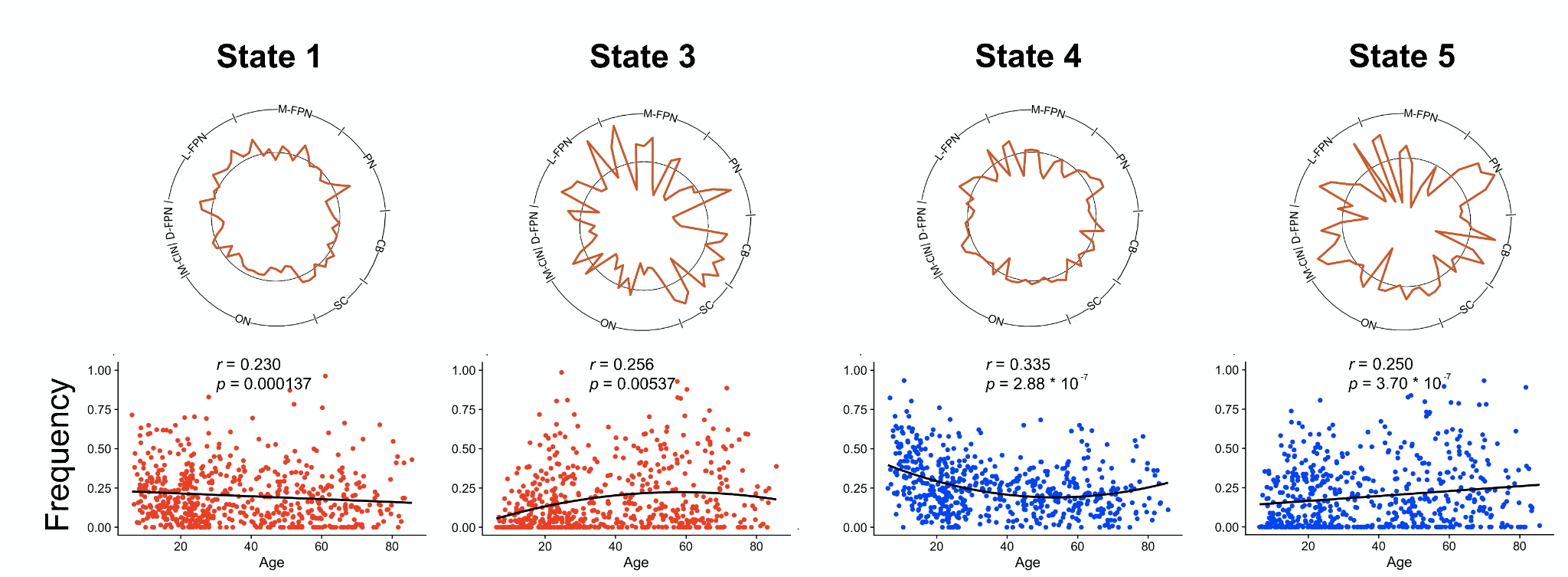


**Supplementary Figure 4.** Associations between state frequency and age using 67.2s windows. Qualitatively identical results to the 44.8s results are shown here: the mostly asynchronous state exhibited a positive quadratic trend; the other low-correlation state exhibited a negative linear trend; the state characterized by sensorimotor, parietal, insular and medial visual brain region synchrony with the salience network exhibited a positive linear trend; and the state characterized by salience network functional connectivity with lateral-frontoparietal, medial-frontoparietal, and subcortical brain regions exhibited a negative quadratic trend.


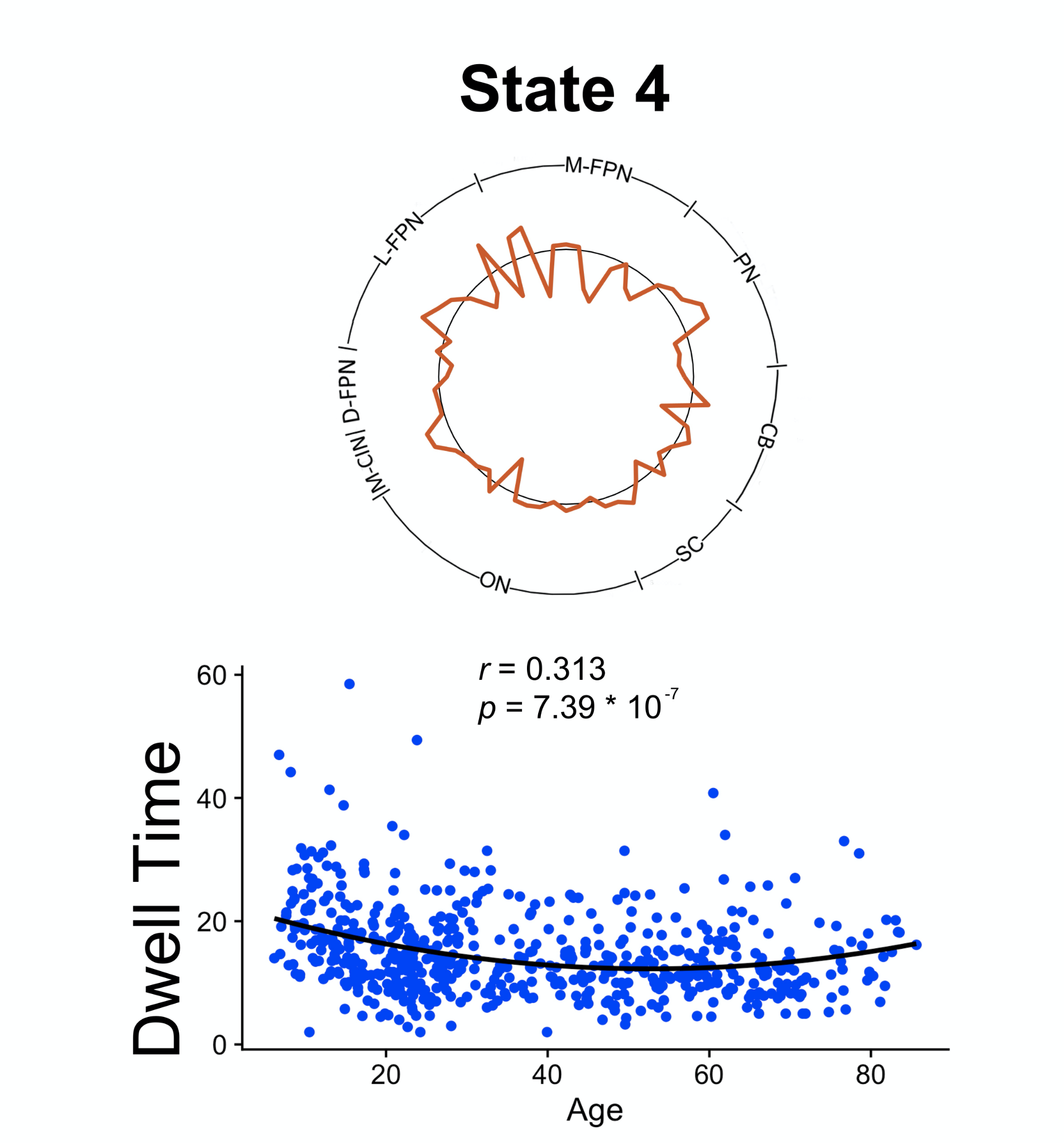


**Supplementary Figure 5.** Associations between state dwell time and age using 67.2s windows. As in the 44.8s results, the state characterized by mostly near-zero correlations with the salience network exhibited a positive quadratic age trend with dwell time.


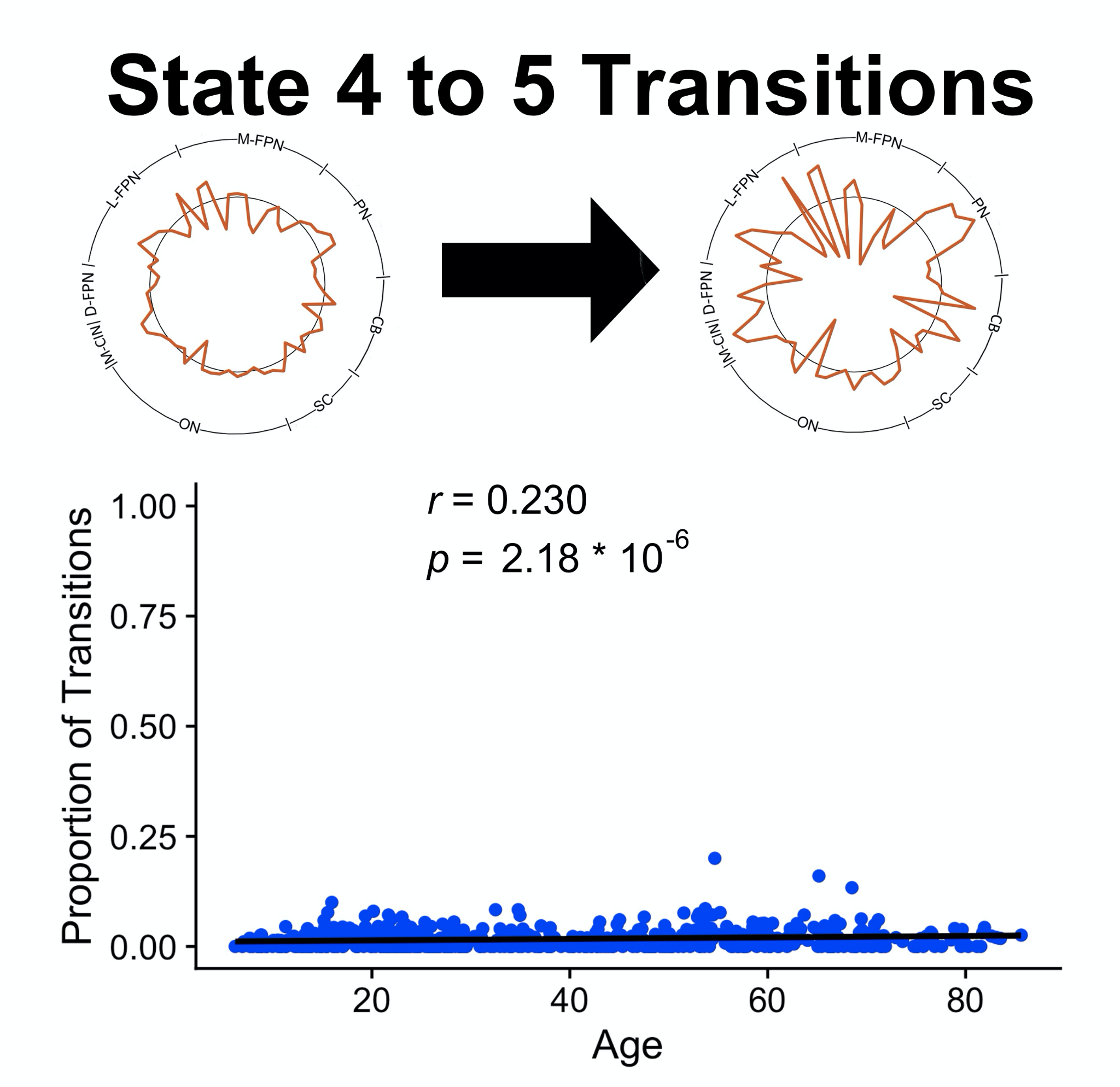


**Supplementary Figure 6.** Associations between state transitions and age using 67.2s windows. As in the 44.8s results, the positive linear trend is observed for the proportion of transitions from the mostly near-zero correlation state to the state characterized by sensorimotor, parietal, insular and medial visual brain region synchrony with the salience network.

**Supplementary Figure 7.** Elbow plot for *k*-means clustering of 89.6s window results.
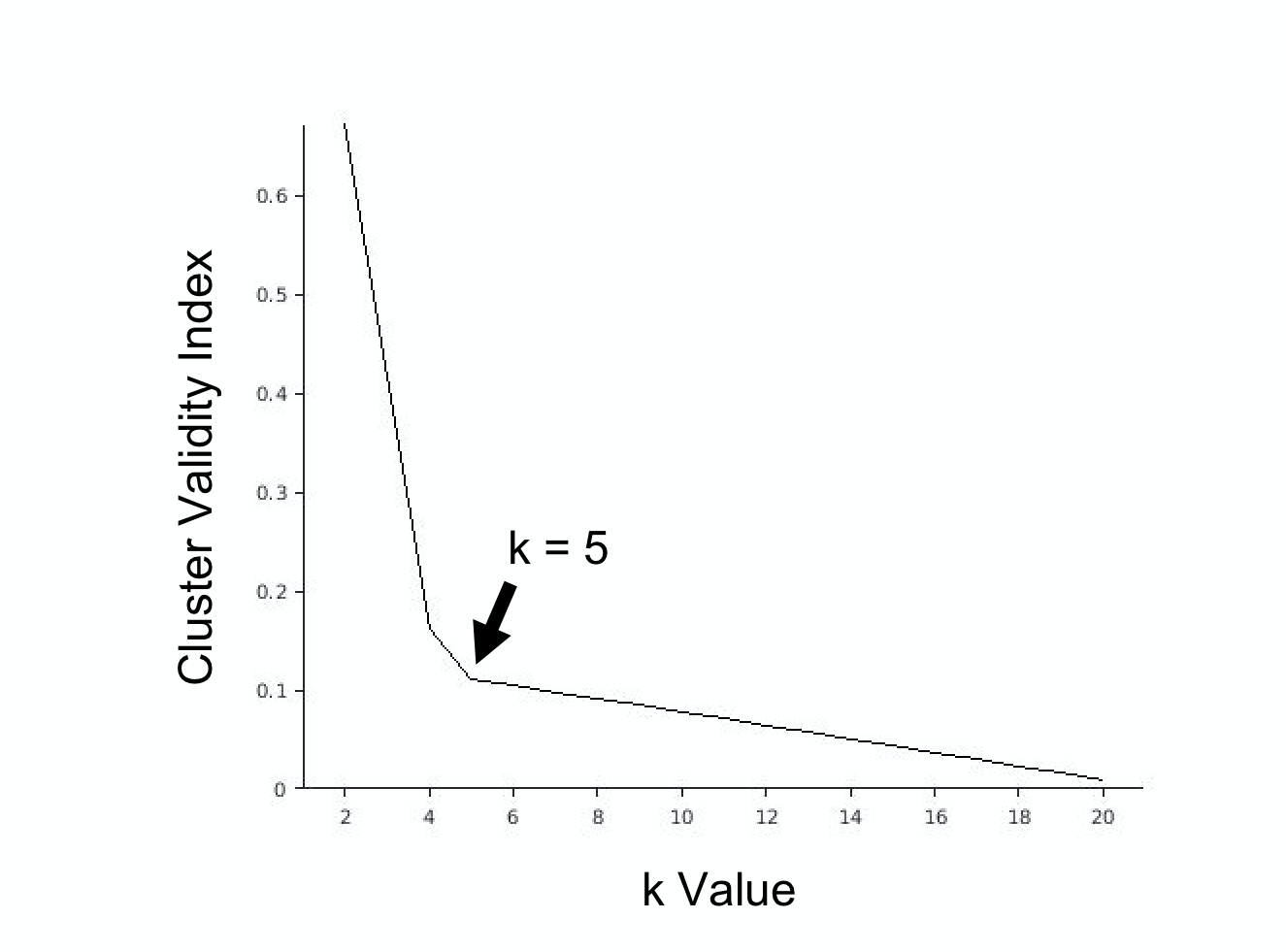


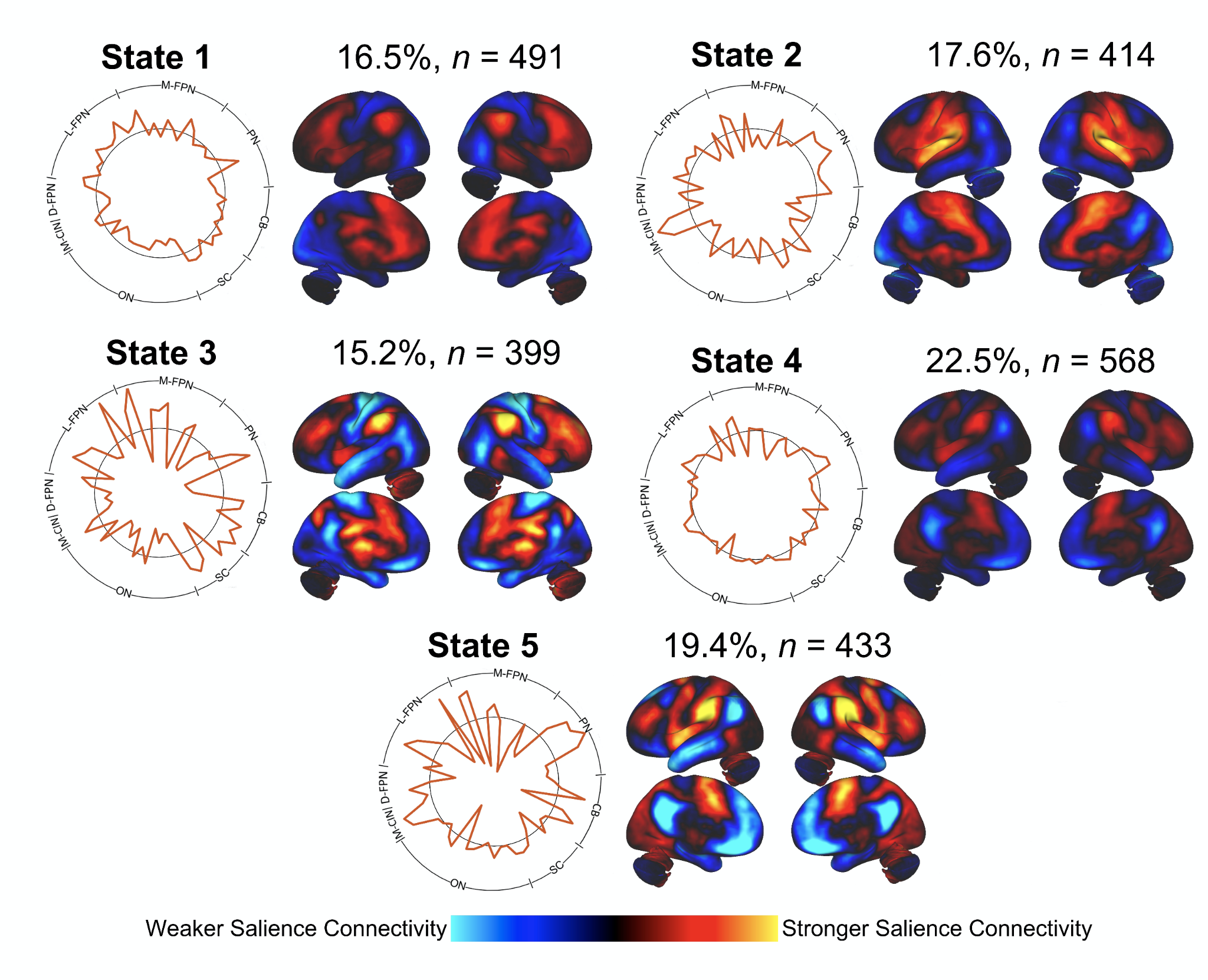


**Supplementary Figure 8.** States of salience network dFC with 89.6 s sliding windows. States revealed were highly similar to those found using 44.8s and 67.2s sliding windows, with two states having low/near-zero correlations and the other states exhibiting the same patterns seen in states using 44.8s and 67.2s windows.

**Supplementary Figure 9.** Associations between state frequency and age using 89.6s windows. Qualitatively identical results to the 44.8s and 67.2s results are shown here: the mostly asynchronous state exhibited a positive quadratic trend; the state characterized by sensorimotor, parietal, insular and medial visual brain region synchrony with the salience network exhibited a positive linear trend; and the state characterized by salience network functional connectivity with lateral-frontoparietal, medial-frontoparietal, and subcortical brain regions exhibited a negative quadratic trend. Notably, however, the other low-correlation state exhibited a negative linear trend in both the 44.8s and 67.2s sliding window results but did not reach significance in the 89.6s window results.
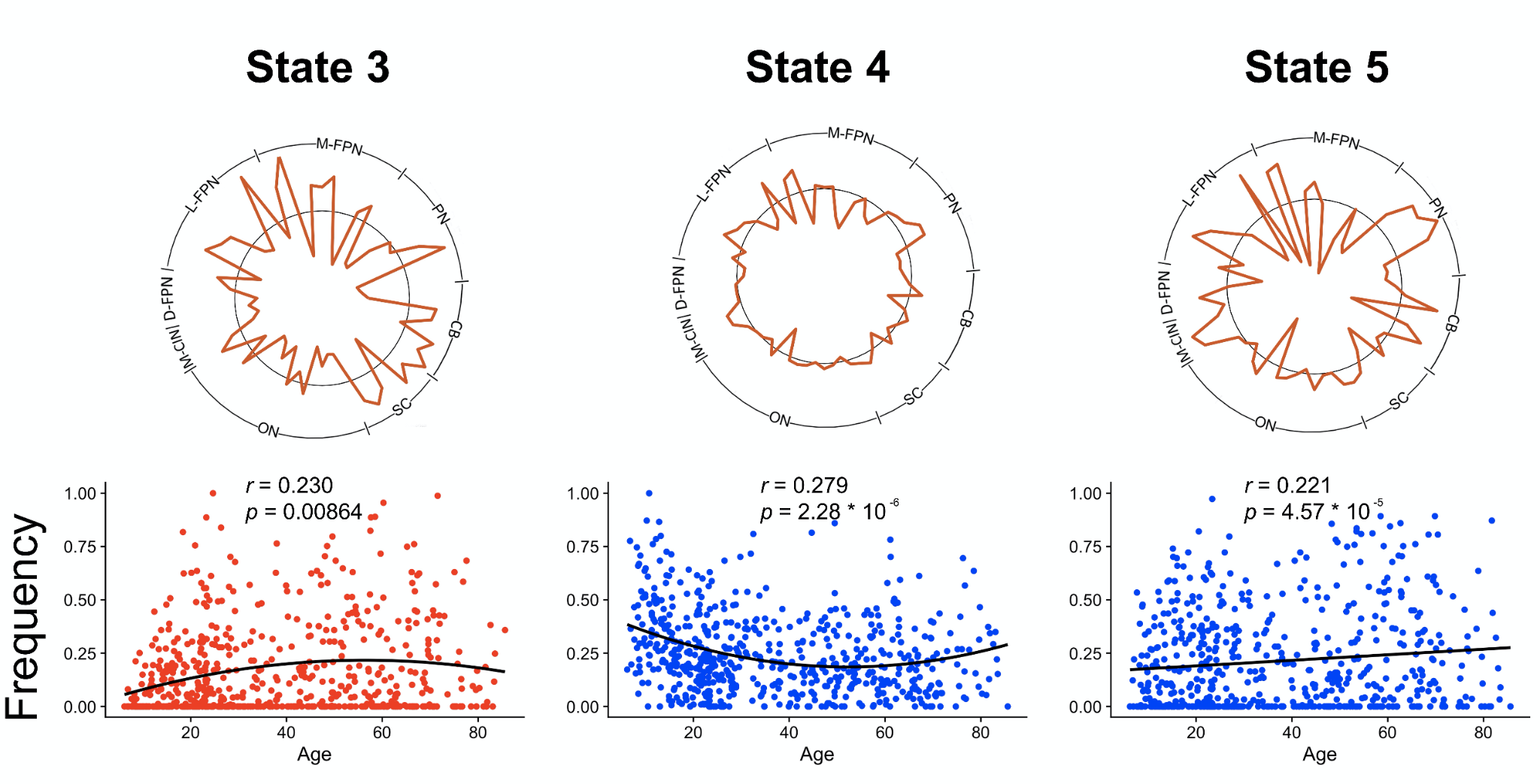


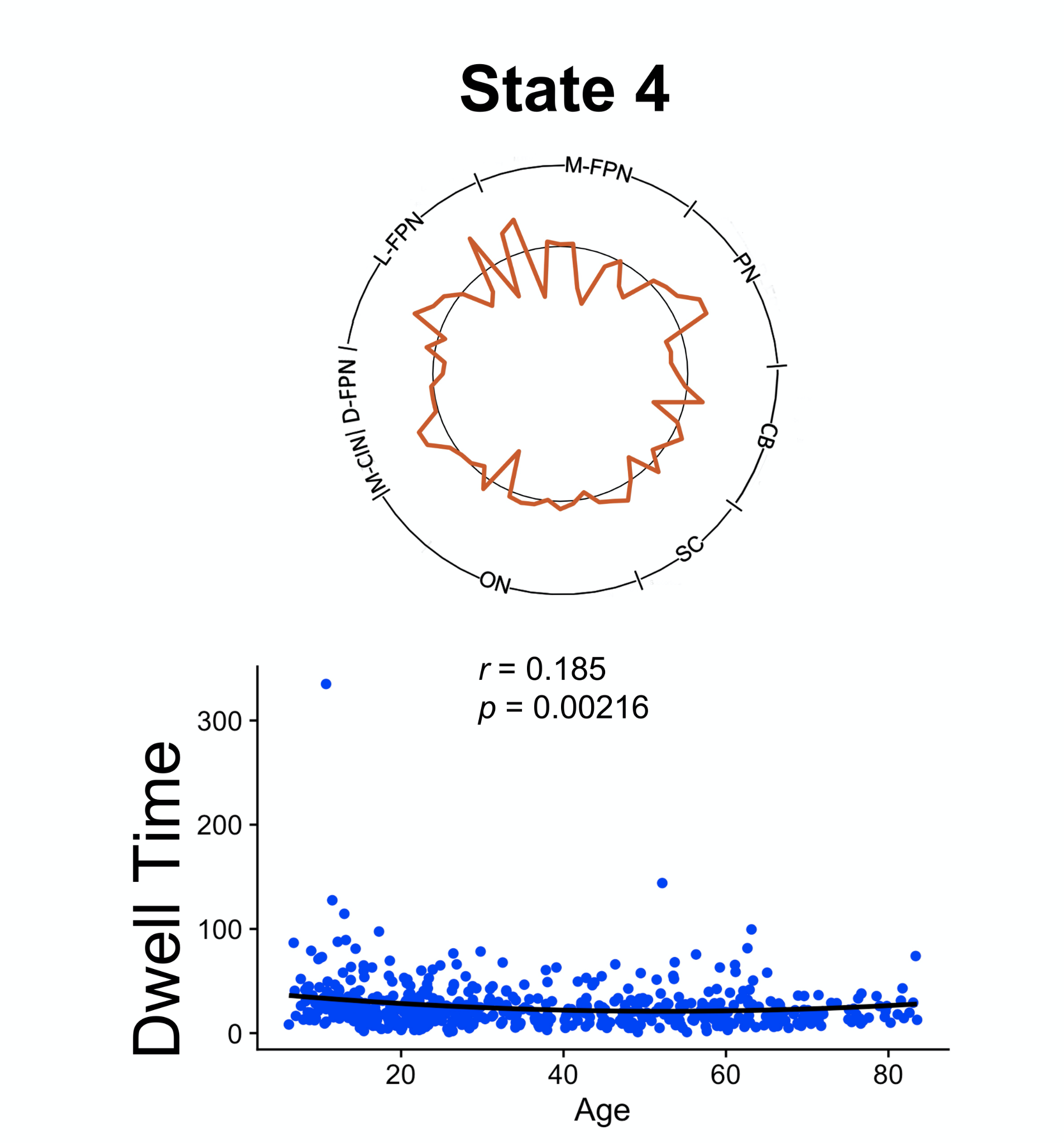


**Supplementary Figure 10.** Associations between state dwell time and age using 89.6s windows. As in the 44.8s and 67.2s results, the state characterized by mostly near-zero correlations with the salience network exhibited a positive quadratic age trend with dwell time.


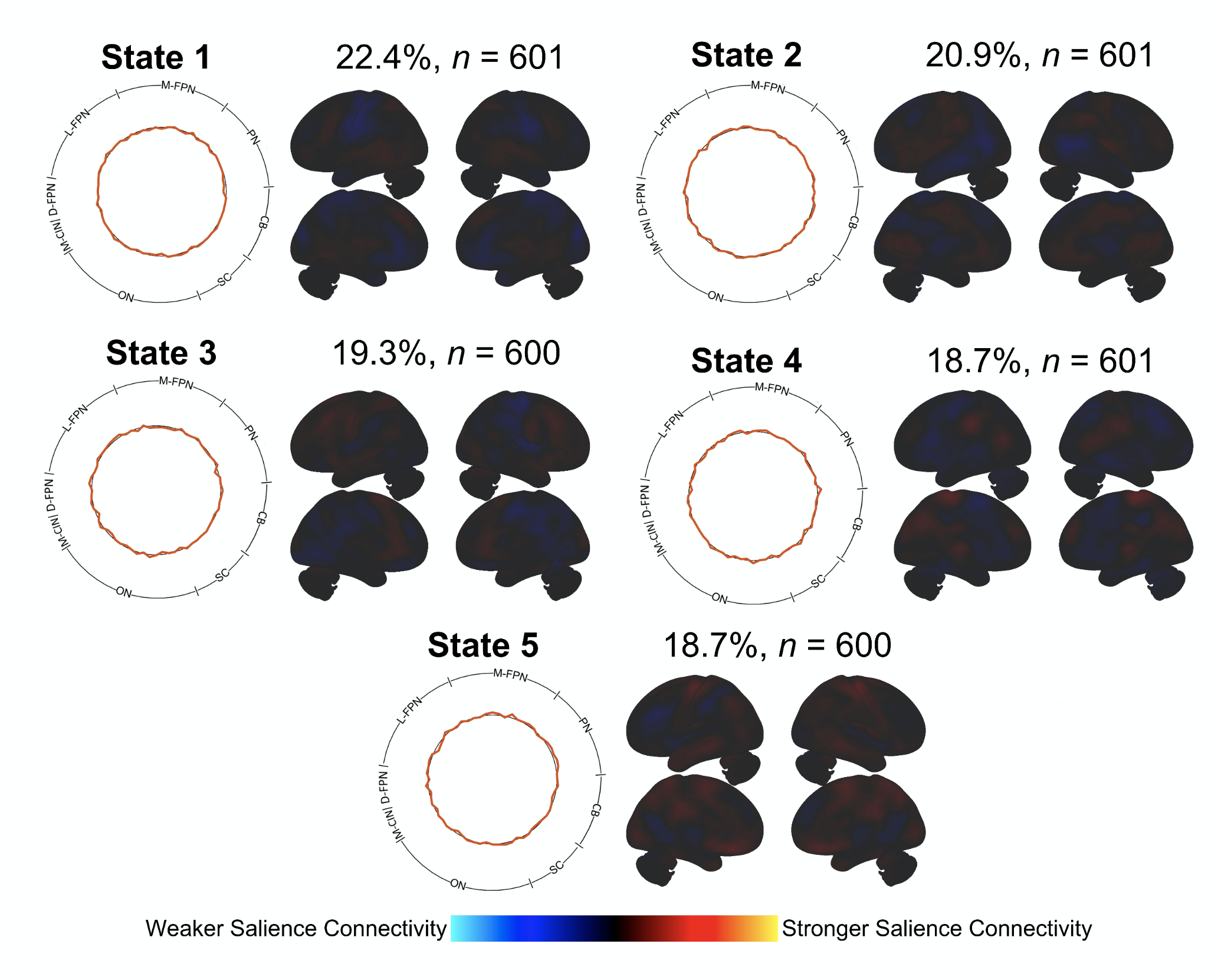


**Supplementary Figure 11.** States of salience network dFC with phase-randomized surrogate data and 44.8s sliding windows. For each state, near-zero correlations between the salience network and all other brain regions were observed. Therefore, no distinct patterns of dFC arise from the processing pipeline under a null model of phase-randomized fMRI time series.
